# A20 FECAL MICROBIOTA TRANSPLANTATION FORMULATIONS DO NOT AFFECT BACTERIAL VIABILITY OR TREATMENT OUTCOMES IN RECURRENT C. DIFFICILE INFECTION

**DOI:** 10.1093/jcag/gwad061.020

**Published:** 2024-02-14

**Authors:** L Chan, R Franz, C McDougall, K Wong, D Kao

**Affiliations:** University of Alberta Department of Medicine, Edmonton, AB, Canada; University of Alberta Division of Gastroenterology, Edmonton, AB, Canada; University of Alberta Division of Gastroenterology, Edmonton, AB, Canada; University of Alberta Division of Gastroenterology, Edmonton, AB, Canada; University of Alberta Division of Gastroenterology, Edmonton, AB, Canada

## Abstract

**Background:**

Fecal microbiota transplantation (FMT) is the most effective therapy for recurrent Clostridioides difficile infections (rCDI) with multiple RCTs showing efficacy of 80-90%. Frozen FMT is a commonly used formulation but requires storage at -80°C, creating barriers to storage and transportation. More recently, lyophilized FMT (LFMT) has shown promise as an alternative formulation, but little data exists as to how bacterial viability may be affected compared to fresh or frozen FMT by different storage temperatures and duration, and real-world outcome data is sparse. As such, this study aims to evaluate the viability and efficacy of LFMT compared to fresh and frozen FMT.

**Aims:**

To 1) compare bacterial viability in 3 FMT formulations: fresh, frozen and lyophilization, at varying storage temperatures and duration, and 2) correlate different formulations with treatment outcomes.

**Methods:**

We compared bacterial viability using flow cytometry to determine the proportion of live vs dead bacteria on fresh, frozen and lyophilized donor stool samples stored at different temperatures (-80°C for frozen, -80°C and +4°C for LFMT) and duration (up to 12 months). We then retrospectively examined treatment success rates in rCDI patients who received fresh, frozen or LFMT at the University of Alberta since inception of the FMT program in 2012. Success is defined as no recurrence of CDI 8 weeks after receiving a single FMT treatment.

**Results:**

Compared to fresh FMT (20% live cells), the proportions of live cells in frozen formulation stored at -80°C and lyophilized formulations stored at -80°C and 4°C did not diminish over time for up to 12 months as shown in Figure 1. Between 2013-2022, outcome data was available in 503 rCDI patients with baseline characteristics and treatment outcomes shown in Table 1. A logistic regression analysis comparing LFMT to frozen FMT resulted in an odds ratio of 0.14, indicating that success rate of LFMT is 14% higher than frozen FMT.

**Conclusions:**

Our study showed that LFMT stored at either -80°C or 4°C over 12 months did not result in diminished bacterial viability compared with frozen FMT. Furthermore, treatment success rate following LFMT in rCDI patients was not lower than with frozen FMT. This finding has implication for stool banks and FMT programs as LFMT can be stored and shipped at 4°C, facilitating use in outpatient settings.

Table 1. Baseline characteristics and success rates of rCDI patients who received fresh, frozen or LFMT.

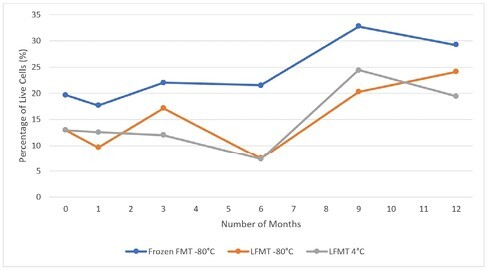

Figure 1. Proportions of live cells in frozen FMT stored at -80°C, LFMT stored at -80°C and 4°C, at 1, 3, 6, 9 and 12 months.

**Funding Agencies:**

None

**Table:** 

	Fresh FMT	Frozen FMT	LFMT
Number of patients	33	440	30
Mean age (SD), years	61 (21)	66 (18)	67 (16)
Female, %	55	59	63
Mean CDI episodes (SD) prior to FMT	3.4 (1.3)	3.5 (1.2)	3.4 (0.9)
Success rate after a single treatment, %	90.9	81.6	83.3
Mean number of doses (SD) given per patient	1.0 (0.2)	1.1 (0.5)	1.0 (0.2)

